# Clinical and microbiological characteristics of men with nonobstructive acute pyelonephritis: A multicenter retrospective observational study

**DOI:** 10.1097/MD.0000000000027386

**Published:** 2021-10-08

**Authors:** Min Gu Park, Sung Yong Cho, Se Yun Kwon, Hoon Choi, Jeong Woo Lee

**Affiliations:** a Department of Urology, Inje University Seoul Paik Hospital, Inje University College of Medicine, Seoul, Korea; b Department of Urology, Inje University Ilsan Paik Hospital, Inje University College of Medicine, Goyang, Korea; c Department of Urology, Dongguk University Gyeongju Hospital, Dongguk University College of Medicine, Gyeongju, Korea; d Department of Urology, Korea University Ansan Hospital, Korea University College of Medicine, Ansan, Korea; e Department of Urology, Kyung Hee University Medical Center, Kyung Hee University College of Medicine, Seoul, Korea.

**Keywords:** antibiotic susceptibility, bacteremia, community-acquired, healthcare-associated, pyelonephritis

## Abstract

To investigate the differences in clinical and microbiological features in men hospitalized with community-acquired (CA) and healthcare-associated (HA) nonobstructive acute pyelonephritis (APN), as well as the predictive factors associated with bacteremia.

Men discharged from urological centers with nonobstructive APN were identified using an electronic medical records system. We compared the clinical and microbiological data between subjects with CA-APN and HA-APN.

Of the 245 men with nonobstructive APN, 175 had CA-APN, and 70 had HA-APN. The HA group was significantly older, had a longer hospital stay, and had more underlying diseases, bacteremia, and intensive care unit admissions than the CA group. The most commonly cultured microorganism was *Escherichia coli.* The susceptibility of the cultured bacteria to fluoroquinolone was 68.7% in the CA group and 45.3% in the HA group (*P* = .005). The proportion of extended-spectrum beta-lactamase-producing bacteria was 22.7% for CA and 53.5% for HA (*P* < .001). The sensitivity to piperacillin/tazobactam was 94.9% for CA and 90.0% for HA, and the sensitivity to amikacin was more than 95% for both groups. The multivariate analysis revealed that an age ≥65 years and chronic liver disease were independent predictive factors for bacteremia.

The incidence of antibiotic resistance and bacteremia was higher in the HA group than in the CA group. However, resistance to fluoroquinolone and the presence of extended-spectrum beta-lactamase-producing bacteria were high in both groups. Piperacillin/tazobactam and amikacin may be suitable treatment options in men with nonobstructive APN.

## 1 Introduction

Acute pyelonephritis (APN) is the most common cause of bacteremia in hospitalized patients.^[1,2]^ Healthcare-associated (HA) APN and community-acquired (CA) APN are known to differ regarding antibiotic resistance^[3]^; however, no previous study has comparatively analyzed HA-APN and CA-APN in male patients. In our previous retrospective study on acute bacterial prostatitis (ABP), significant differences were observed in etiology and antibiotic resistance between CA-ABP and HA-ABP.^[4,5]^ APN can be classified according to the presence of obstruction of the urinary tract into obstructive APN, which is accompanied by hydronephrosis, and nonobstructive APN. Only a few studies have examined nonobstructive APN exclusively, and no studies of nonobstructive APN have been performed in men.

A Korean epidemiological study reported that APN is rare in men, with the incidence of APN in women being 11 times higher than that of men, but that the prevalence of APN is increasing overall.^[6]^ In 1 study that compared the etiology of sepsis and treatment outcomes in 1192 patients admitted to the intensive care unit (ICU), male patients tended to have a higher disease severity and mortality rate than female patients, and although urosepsis was more common in women, hospital mortality rates were significantly higher in men.^[7]^ Understanding the features of APN in men is important, given its increasing incidence and more serious outcomes in men. Similarly, considering the consistent rise in antibiotic resistance related to APN worldwide,^[8,9]^ selecting appropriate empirical antibiotics in male patients with APN is crucial, as these patients are highly likely to present to the hospital in a more serious condition, even early on in the disease course.

Based on this background, we aimed to investigate the differences in the clinical and microbiological features in men who are hospitalized with nonobstructive CA-APN and HA-APN, as well as predictive factors associated with bacteremia. We hypothesized that differences would be observed between HA-APN and CA-APN in men.

## 2 Patients and methods

### 2.1 Patients

We performed a multicenter retrospective observational study. Men (>20 years) with nonobstructive APN as a discharge diagnosis from urological centers of 5 university hospitals in the Republic of Korea were identified from January 2011 to December 2017 using an electronic medical records system. We surveyed patients’ age, body mass index (BMI), underlying diseases, urinary tract abnormalities, urinary catheter and percutaneous nephrostomy, ureteral catheter insertion, complete blood count, C-reactive protein levels, results of urine and blood culture (including antibiotic sensitivity results and the existence of extended-spectrum beta-lactamase (ESBL)-producing bacteria), length of hospital stay, length of ICU stay, and mortality. Patients with anatomical obstruction, such as ureteropelvic junction obstruction and ureterovesical obstruction, anatomical obstruction due to other causes, such as urinary tract stone and tumor, as well as patients with functional obstruction, such as acute urinary retention and neurogenic bladder (including APN related to Foley catheter or clean intermittent catheterization), were excluded.

### 2.2 Diagnosis and classification of APN

APN should be considered as a diagnosis in the presence of 1 or more of the following findings^[10]^: a body temperature of 38 °C or higher, accompanied by flank pain, costovertebral angle tenderness, or lower urinary tract symptoms, with at least 5 leukocytes per high power field on urine microscopy. Finally, nonobstructive APN was diagnosed by abdominal computed tomography scan.

APN was also classified as either HA or CA. HA-APN was defined according to the classification by Friedman et al as follows^[11]^: receiving intravenous therapy, wound care, or specialized nursing care at home 30 days before APN; visiting a hemodialysis center or received intravenous chemotherapy within 30 days of presentation; received inpatient treatment at an acute care hospital for at least 2 days within the previous 3 months before APN; residing in a nursing home or long-term care facility; or received invasive urinary procedure, urological surgery, or urethral catheterization within 30 days of developing APN.

Patients with APN who did not fulfill any of the above-mentioned criteria were defined as CA-APN patients.

### 2.3 Microbiological data

ESBL are enzymes that give resistance to most beta-lactam antibiotics, including penicillins, cephalosporins, and the monobactam aztreonam. Bacteremia is defined when there are bacteria present in bloodstream. The causative microbes were confirmed by the presence of microorganisms with a colony count of 10^5^ CFU/mL in urine cultures or the isolation of microorganisms from blood specimens of patients using automated methods. Detection of ESBL producing *Escherichia coli* and antibiotic susceptibility patterns of urinary tract pathogens were identified using a Vitek II automated system (bioMérieux Inc.) or Microscan (Siemens Healthcare Inc.) at each hospital. The susceptibility of a uropathogen to an antibiotic was determined by interpreting the breakpoints recommended by the Clinical and Laboratory Standards Institute (CLSI) guidelines (2017).^[12]^ Microbial isolates showing intermediate antimicrobial susceptibility were considered to be resistant.

### 2.4 Statistical analysis

The 2 groups were compared using independent *t* tests, and predictors of bacteremia were identified using univariate and multivariate analyses with logistic regression models. Statistical significance was defined as a 2-tailed *P* value of < .05. Statistical analyses were performed with IBM SPSS Statistics for Windows, version 20.0 (IBM Corp., Armonk, NY).

### 2.5 Ethics statement

All investigations and analyses in studies involving medical data of patients were in accordance with the ethical standards of the institutional research review board and with the 1964 Helsinki declaration and its later amendments or comparable ethical standards. Informed consent was not required due to the retrospective nature of this study.

## 3 Results

Of the 245 men with nonobstructive APN, 175 had CA-APN, and 70 had HA-APN. The mean age of the 245 patients was 61.5 ± 17.4 years, and the mean BMI was 22.9 ± 3.6 kg/m^2^, with the HA group having a significantly lower BMI than the CA group. Regarding clinical characteristics and laboratory findings, the HA group was significantly older and had a higher incidence of underlying diseases than the CA group. The incidence of bacteremia was significantly higher in the HA group (35.7%) than in the CA group (18.9%), and the percentage of ICU admissions (25.7% vs 3.4%) and length of hospital stay (18.4 vs 8.3 days) also significantly differed between groups. The mortality rate was higher in the HA group (4.3%) than in the CA group (1.7%); however, this difference was insignificant (Table 1).

**Table 1:** Clinical characteristics and laboratory findings of men with nonobstructive acute pyelonephritis.

Variable	Total (n = 245)	Community-acquired (n = 175)	Healthcare-associated (n = 70)	*P*
Age (yrs)	61.5 ± 17.4	58.1 ± 17.5	70.0 ± 14.0	<.001
BMI (kg/m^2^)	22.9 ± 3.6	23.3 ± 3.6	22.1 ± 3.5	.023
Underlying disease				
Cardiovascular disease	116 (47.3)	71 (40.6)	45 (64.3)	.001
Diabetes mellitus	83 (33.9)	51 (29.1)	32 (45.7)	.013
Neurologic disease	69 (28.2)	25 (14.3)	44 (62.9)	<.001
Solid tumor	22 (9.0)	9 (5.1)	13 (18.6)	.001
Chronic kidney disease	19 (7.8)	14 (8.0)	5 (7.1)	1.000
Chronic pulmonary disease	16 (6.5)	7 (4.0)	9 (12.9)	.011
Chronic liver disease	14 (5.7)	9 (5.1)	5 (7.1)	.550
Pancreaticobiliary disease	4 (1.6)	3 (1.7)	1 (1.4)	1.000
Hematologic disease	3 (1.2)	0 (0.0)	3 (4.3)	.023
WBC ×10^3^/μL	13.48 ± 5.7	13.18 ± 5.3	14.23 ± 6.6	.191
ANC ×10^3^/μL	10.90 ± 5.5	10.54 ± 5.0	11.79 ± 6.4	.105
CRP (mg/dL)	12.0 ± 8.2	11.3 ± 8.6	13.6 ± 7.0	.055
Bacteremia	58 (23.7)	33 (18.9)	25 (35.7)	.005
ICU care	24 (9.8)	6 (3.4)	18 (25.7)	<.001
Duration of hospital stay (d)	11.2 ± 11.8	8.3 ± 7.6	18.4 ± 16.5	<.001
Mortality	6 (2.4)	3 (1.7)	3 (4.3)	.228

Continuous variables are shown as mean standard ± deviation and categorical variables as number (%). ANC = absolute neutrophil count, BMI = body mass index, CRP= C-reactive protein, ICU = intensive care unit, WBC = white blood cell.

Bacterial culture was performed in 154 of the 245 patients (62.9%), with significant differences between the HA and CA groups (HA: 75.7%, CA: 57.7%, *P* = .044). Commonly cultured microorganisms included *E coli* (41.7% and 50.0% in the CA and HA groups, respectively, Table 2).

**Table 2:** Microbiological examination in men with nonobstructive acute pyelonephritis.

Variable	Total (n = 245)	Community-acquired (n = 175)	Healthcare-associated (n = 70)	*P* = .044
Sterile	91 (37.1)	74 (42.3)	17 (24.3)	
*Escherichia coli*	108 (44.1)	73 (41.7)	35 (50.0)	
*Enterococci* spp.	13 (5.3)	11 (6.3)	2 (2.7)	
*Klebsiella* spp.	10 (4.1)	5 (2.9)	5 (7.1)	
*Pseudomonas* spp.	5 (2.0)	2 (1.1)	3 (4.3)	
*Enterobacter* spp.	4 (1.6)	2 (1.1)	2 (2.9)	
Coagulase-negative staphylococci	4 (1.6)	3 (1.7)	1 (1.4)	
*Staphylococcus aures*	2 (0.8)	2 (1.1)	0 (0.0)	
*Serratia marcescens*	1 (0.4)	0 (0.0)	1 (1.4)	
*Acinetobacter* spp.	1 (0.4)	0 (0.0)	1 (1.4)	
*Streptococcus agalactiae*	1 (0.4)	1 (1.1)	0 (0.0)	
*Aerococcus urinae*	1 (0.4)	0 (0.0)	1 (1.4)	
Mixed	4 (1.6)	2 (1.1)	2 (2.9)	

The susceptibility of the cultured bacteria to fluoroquinolone was 68.7% in the CA group and 45.3% in the HA group, with a significant difference between the 2 groups. The proportion of ESBL-producing bacteria was 22.7% and 53.5% in the CA and HA groups, respectively, and the differences between the 2 groups were significant. In the CA and HA groups, the sensitivity to piperacillin/tazobactam was 94.9% and 90.0%, respectively, with no significant differences between groups. Amikacin showed more than 95% sensitivity to bacteria isolated from both groups, and there were no significant differences between the 2 groups. Similarly, susceptibility to imipenem was high in both groups (CA: 97.7%, HA: 95.8%), with no significant differences between groups (Table 3).

**Table 3:** Antimicrobial susceptibility of bacteria isolated from men with nonobstructive acute pyelonephritis.

	Community-acquired (n = 175)			Healthcare-associated (n = 70)			
	Total	Susceptible	Susceptibility (%)	Total	Susceptible	Susceptibility (%)	*P*
Fluoroquinolone	99	68	68.7	53	24	45.3	.005
Amikacin	79	78	98.7	47	45	95.7	.555
Gentamicin	86	59	68.6	49	31	63.3	.527
TMP-SMX	91	63	69.2	46	31	67.4	.827
Ampicillin	92	34	37.0	48	13	27.1	.240
Amoxicillin/CA	78	61	78.2	40	26	65.0	.123
Piperacillin/Tazobactam	79	75	94.9	48	43	90.0	.297
Imipenem	87	85	97.7	48	46	95.8	.616
ESBL-producing	88	20^∗^	22.7^†^	43	23^∗^	53.^5^^†^	.000

CA = clavulanic acid, ESBL = extended-spectrum beta-lactamase, TMP-SMX = trimethoprim-sulfamethoxazole.

∗ The number of ESBL-producing bacteria.

† The percentage of ESBL-producing bacteria.

In the logistic regression performed to identify the predictors of bacteremia, multivariate analysis revealed that age ≥65 years and chronic liver disease were independent predictive factors for bacteremia (Table 4).

**Table 4:** Univariate and multivariate analysis for clinical factors related to bacteremia in men with nonobstructive acute pyelonephritis.

	B	S.E.	*P* value	Exp (B)	95% CI
Univariate analysis					
Age (≥65 yrs)	−1.681	0.250	<.001	0.186	1.326-4.576
BMI (>25 kg/m^2^)	0.016	0.345	.963	1.016	0.517-1.999
Underlying disease					
Cardiovascular disease	0.503	0.303	.097	1.654	0.913-2.997
Diabetes mellitus	0.522	0.309	.091	1.685	0.920-3.086
Neurologic disease	0.801	0.317	.011	2.227	1.197-4.144
Solid tumor	0.682	0.471	.148	1.977	0.785-4.981
Chronic kidney disease	0.694	0.502	.166	2.002	0.749-5.349
Chronic liver disease	0.948	0.563	.092	2.582	0.857-7.776
Chronic pulmonary disease	0.714	0.540	.186	2.042	0.709-5.885
Healthcare-associated APN	0.872	0.316	.006	2.391	1.288-4.437
Multivariate analysis					
Age (≥65 yrs)	0.737	0.365	.043	2.089	1.022-4.272
Cardiovascular disease	0.092	0.348	.790	1.097	0.555-2.168
DM	0.274	0.339	.419	1.315	0.677-2.554
Neurologic disease	0.421	0.381	.269	1.523	0.722-3.212
Chronic liver disease	1.315	0.603	.029	3.726	1.142-12.154
Hospital-acquired APN	0.351	0.379	.354	1.421	0.675-2.989

Univariate and multivariate analyses were performed using logistic regression models. APN = acute pyelonephritis, BMI = body mass index.

## 4 Discussion

The results of this study showed that the HA-APN group had a significantly higher incidence of bacteremia, rate of ICU admissions, and longer length of hospital stay than the CA-APN group. Although statistically insignificant, the mortality rate was also higher in the HA-APN group than in the CA-APN group. Therefore, that male patients who develop HA-APN may show a more serious clinical course. Additionally, our previous study on ABP showed that the development of bacteremia was significantly higher after a prostate biopsy in HA-ABP cases than in CA-ABP cases.^[4]^ Similarly, Ha et al^[13]^ and Kim et al^[14]^ reported that HA-ABP had a significantly lower sensitivity to antibiotics, such as quinolone, a higher probability of ESBL-producing bacteria, and a more serious clinical course than CA-ABP.

Predicting and preparing for the possibility of progression to septic shock is important in the treatment and management of APN. Studies on patients who developed septic shock found a higher mortality rate in men than in women.^[7]^ Multiple logistic regression analysis was performed to identify the risk factors that could explain the differences in the 28-day mortality and in-hospital mortality between men and women, and urinary tract infection (UTI) was identified as a significant predictor.^[7]^ Therefore, it should be noted that male patients are likely to show a poorer clinical course than female patients in UTI sepsis. In fact, in a study on the prevalence of APN in Korea, Kim et al reported that APN mortality rate in Korea is 1.5 per 1000 cases in men, which is higher than 0.5 per 1000 cases in women, with a longer hospital stay observed in men (10.5 days) than in women (8.9 days).^[6]^ In our study, the mortality rate was 2.4% (6/245), which was substantially higher than the general mortality rate of APN among Korean men, and the mortality rate of HA-APN was even higher at 4.3% (3/70). Kim et al reported that the actual APN mortality rate is estimated to range between 0.6% and 1.3% due to the possibility that in severe or fatal cases, sepsis, rather than APN, would have been the primary diagnosis in their study.^[6]^ The mortality rate of nonobstructive APN in men in our study is still considerably high compared to 1.3 per 1000 cases. Similarly, the mean length of hospital stay was longer in our study (11.2 days) than the 10.5 days reported for APN in Korean men.^[6]^ In particular, the HA-APN group had a significantly longer hospital stay (18.4 days) than the CA-APN group. These results suggest that the severity of nonobstructive APN in men is quite high and that a long-term hospital stay with a possibility of high mortality should be considered promptly in the treatment of HA-APN.

A Korean epidemiological study showed that the incidence of APN is continuously increasing in both men and women. Although there is a possibility that the virulence of *E coli* might have changed, it is surmised that changes in the *E coli* strain have contributed to the rising incidence.^[6]^ In our study, *E coli* was the most common bacterium identified, and the incidence was higher in the HA-APN group than in the CA-APN group. The ST131 clone of *E coli*, which is notorious for antimicrobial resistance and extensive virulence profile, has increased in both CA- and HA-UTIs worldwide.^[15–17]^ In Korea, the proportion of ST131 strain isolated from CA *E coli* bacteremia, which was 19.7% (15/76) from 2006 to 2011, increased to 26.9% (32/119) in 2016.^[18,19]^ Similarly, infections caused by ESBL-producing *E coli* have been increasing worldwide, and the urinary tract is 1 of the most common infection sites.^[20]^ A high Charlson co-morbidity score, history of admissions, and previous antibiotic use are known risk factors for APN caused by ESBL pathogens.^[21]^ ESBL-producing *E coli* is also related to long hospitalization stays and recurrent UTI.^[22,23]^ The percentage of ESBL-producing bacteria in our study was 22.7% in the CA-APN group, which is substantially higher than that reported for nonobstructive CA-APN in women (5.5-9.0%).^[24]^ The HA-APN group showed more than a 2-fold increase in ESBL-producing bacteria compared to the CA-APN group, which is likely to have influenced the significantly higher rate of ICU care and bacteremia incidence, as well as longer hospital stay, and higher mortality.

In our study, although liver disease was identified as a significant predictor of bacteremia, the number of patients with liver disease was rather small. According to Burroughs et al^[25]^, patients with primary biliary cirrhosis are at increased risk of developing UTI, and 4 case-control studies have confirmed an association between increased UTI risk and primary biliary cirrhosis.^[26–29]^ A study that analyzed patients with septic shock caused by bacteremia found that multiple organ failure was more predominant in men than in women, with men showing a significantly greater incidence of kidney, respiratory, and liver dysfunctions as well as metabolic failure, than women.^[7]^ Considering our findings, clinicians should be prepared for the possibility of patients with liver dysfunction developing bacteremia-induced septic shock. Further, Chang et al^[24]^ reported a significantly longer duration of intravenous antibiotics treatment and hospital stay in the elderly group (age ≥65 years) than in the non-elderly group in their study of nonobstructive CA-APN in women. In addition, Park et al^[7]^ observed an increasing 28-day mortality and hospital mortality with advancing age in both men and women in their study of patients with CA severe sepsis and septic shock. Therefore, clinicians should be thoroughly prepared for bacteremia-induced septic shock in elderly male patients with APN.

In these patients, empirical antibiotic treatment is a critical issue. Our results showed that susceptibility to fluoroquinolones was substantially diminished to below 70%. Particularly, the HA-APN group showed a lower rate of antibiotic susceptibility than the CA-APN group. This was even lower than that found among female patients with nonobstructive APN (80.6-85.2%).^[24]^ Choosing a fluoroquinolone as the first-line antibiotic is likely to lead to a poor clinical outcome in older patients or patients with liver disease. Thus, in these patients, piperacillin/tazobactam or amikacin should be considered as first-line antibiotics in both CA-APN and HA-APN in men. In high-risk patients or those with severe diseases, carbapenems, such as imipenem, should be immediately selected for treatment.

This study has a few limitations. Due to the retrospective design, some patients were excluded because of incomplete data and the absence of follow-up. In addition, details of preadmission antibiotic therapy, including the type of antibiotics used as well as a history of UTI, have not been assessed thoroughly. Due to missing information, some patients were wrongly classified as CA-APN when they should be considered HA-APN. We integrated data from 5 centers, and there may have been differences in the methods of culture and inpatient treatment modalities across the hospitals. Finally, the small sample size may have hindered drawing statistically significant conclusions.

Nevertheless, this study is clinically significant as it examined the clinical characteristics of nonobstructive APN in men, a phenomenon that has rarely been studied so far. Prospectively studying a larger sample using a unified protocol would produce a better understanding of the features of nonobstructive APN in men.

## 5 Conclusions

Older men and those with comorbidities, such as liver disease, are more likely to develop bacteremia with a poor clinical course in nonobstructive APN. The HA-APN group showed a higher incidence of antibiotic resistance and bacteremia than the CA-APN group. Therefore, long-term hospital stay and ICU care should be considered promptly in the treatment of HA-APN cases. Resistance to fluoroquinolones and the presence of ESBL-producing bacteria was high in men with nonobstructive APN. Piperacillin/tazobactam and amikacin may be suitable options as empirical antibiotic therapy regardless of disease severity.

## Acknowledgments

We would like to thank Editage (www.editage.co.kr) for editing this manuscript for English language.

## Author contributions

**Conceptualization:** Min Gu Park, Sung Yong Cho, Se Yun Kwon, Hoon Choi, Jeong Woo Lee.

**Data curation:** Min Gu Park, Sung Yong Cho, Se Yun Kwon, Hoon Choi, Jeong Woo Lee.

**Formal analysis:** Jeong Woo Lee.

**Funding acquisition:** Jeong Woo Lee.

**Investigation:** Min Gu Park, Jeong Woo Lee.

**Methodology:** Min Gu Park, Jeong Woo Lee.

**Supervision:** Jeong Woo Lee.

**Writing – original draft:** Min Gu Park.

**Writing – review & editing:** Jeong Woo Lee.

## References

[1] Esposito AL Gleckman RA Cram S. Community-acquired bacteremia in the elderly: analysis of one hundred consecutive episodes. J Am Geriatr Soc 1980;28:315–9. 6993540 10.1111/j.1532-5415.1980.tb00622.x

[2] Gleckman R Blagg N Hibert D. Acute pyelonephritis in the elderly. South Med J 1982;75:551–4. 7079812 10.1097/00007611-198205000-00011

[3] Hyun M Lee JY Kim HA. Comparison of *Escherichia coli* and *Klebsiella pneumoniae* acute pyelonephritis in Korean patients. Infect Chemother 2019;51:130–41. 31270992 10.3947/ic.2019.51.2.130 PMC6609746

[4] Park MG Cho MC Cho SY. Comparison of antibiotic susceptibility of *Escherichia coli* between community-acquired and post-prostate biopsy acute bacterial prostatitis. Arch Esp Urol 2019;72:1018–25. 31823850

[5] Park MG Cho MC Cho SY. Clinical and microbiological features and factors associated with fluoroquinolone resistance in men with community-acquired acute bacterial prostatitis. Urol Int 2016;96:443–8. 26943143 10.1159/000444763

[6] Kim B Myung R Kim J. Descriptive epidemiology of acute pyelonephritis in Korea 2010-2014: Population-based Study. J Korean Med Sci 2018;33:e310. 30505253 10.3346/jkms.2018.33.e310 PMC6262185

[7] Park DW Chun BC Kim JM. Epidemiological and clinical characteristics of community-acquired severe sepsis and septic shock: a prospective observational study in 12 university hospitals in Korea. J Korean Med Sci 2012;27:1308–14. 23166410 10.3346/jkms.2012.27.11.1308 PMC3492663

[8] Zhanel GG Hisanaga TL Laing NM. Antibiotic resistance in *Escherichia coli* outpatient urinary isolates: final results from the North American Urinary Tract Infection Collaborative Alliance (NAUTICA). Int J Antimicrob Agents 2006;27:468–75. 16713191 10.1016/j.ijantimicag.2006.02.009

[9] Sanchez GV Master RN Karlowsky JA. In vitro antimicrobial resistance of urinary *Escherichia coli* isolates among U.S. outpatients from 2000 to 2010. Antimicrob Agents Chemother 2012;56:2181–3. 22252813 10.1128/AAC.06060-11 PMC3318377

[10] Kunin CM. Definition of acute pyelonephritis vs the urosepsis syndrome. Arch Intern Med 2003;163:2393–4. 2393; author reply. 10.1001/archinte.163.19.2393-b 14581259

[11] Friedman ND Kaye KS Stout JE. Health care-associated bloodstream infections in adults: a reason to change the accepted definition of community-acquired infections. Ann Intern Med 2002;137:791–7. 12435215 10.7326/0003-4819-137-10-200211190-00007

[12] Clinical and Laboratory Standards Institute (CLSI). Performance standards for antimicrobial susceptibility testing. 27th ed. Wayne, PA: Clinical and Laboratory Standards; 2017.

[13] Ha US Kim ME Kim CS. Acute bacterial prostatitis in Korea: clinical outcome, including symptoms, management, microbiology and course of disease. Int J Antimicrob Agents 2008;31: Suppl 1: S96–101. 18065208 10.1016/j.ijantimicag.2007.07.041

[14] Kim JW Oh MM Bae JH. Clinical and microbiological characteristics of spontaneous acute prostatitis and transrectal prostate biopsy-related acute prostatitis: is transrectal prostate biopsy-related acute prostatitis a distinct acute prostatitis category? J Infect Chemother 2015;21:434–7. 25701308 10.1016/j.jiac.2015.01.014

[15] Day MJ Doumith M Abernethy J. Population structure of *Escherichia coli* causing bacteraemia in the UK and Ireland between 2001 and 2010. J Antimicrob Chemother 2016;71:2139–42. 27150395 10.1093/jac/dkw145 PMC4954928

[16] Nicolas-Chanoine MH Bertrand X Madec JY. *Escherichia coli* ST131, an intriguing clonal group. Clin Microbiol Rev 2014;27:543–74. 24982321 10.1128/CMR.00125-13 PMC4135899

[17] Johnson JR Porter S Thuras P. The pandemic H30 subclone of sequence type 131 (ST131) as the leading cause of multidrug-resistant *Escherichia coli* infections in the United States (2011-2012). Open Forum Infect Dis 2017;4:ofx089. 28638846 10.1093/ofid/ofx089 PMC5473367

[18] Kim YA Kim JJ Kim H. Community-onset extended-spectrum-beta-lactamase-producing *Escherichia coli* sequence type 131 at two Korean community hospitals: the spread of multidrug-resistant *E. coli* to the community via healthcare facilities. Int J Infect Dis 2017;54:39–42. 27865830 10.1016/j.ijid.2016.11.010

[19] Kang CI Cha MK Kim SH. Clinical and molecular epidemiology of community-onset bacteremia caused by extended-spectrum beta-lactamase-producing *Escherichia coli* over a 6-year period. J Korean Med Sci 2013;28:998–1004. 23853481 10.3346/jkms.2013.28.7.998 PMC3708098

[20] Kang CI Song JH Chung DR. Risk factors and treatment outcomes of community-onset bacteraemia caused by extended-spectrum beta-lactamase-producing *Escherichia coli*. Int J Antimicrob Agents 2010;36:284–7. 20580534 10.1016/j.ijantimicag.2010.05.009

[21] Calbo E Romani V Xercavins M. Risk factors for community-onset urinary tract infections due to *Escherichia coli* harbouring extended-spectrum beta-lactamases. J Antimicrob Chemother 2006;57:780–3. 16492721 10.1093/jac/dkl035

[22] Alevizakos M Nasioudis D Mylonakis E. Urinary tract infections caused by ESBL-producing Enterobacteriaceae in renal transplant recipients: a systematic review and meta-analysis. Transplant Infect Dis 2017;19:e12759. 10.1111/tid.12759 28803446

[23] Kim B Kim J Seo MR. Clinical characteristics of community-acquired acute pyelonephritis caused by ESBL-producing pathogens in South Korea. Infection 2013;41:603–12. 23504297 10.1007/s15010-013-0441-z

[24] Chang UI Kim HW Noh YS. A comparison of the clinical characteristics of elderly and non-elderly women with community-onset, non-obstructive acute pyelonephritis. Korean J Intern Med 2015;30:372–83. 25995668 10.3904/kjim.2015.30.3.372 PMC4438292

[25] Burroughs AK Rosenstein IJ Epstein O. Bacteriuria and primary biliary cirrhosis. Gut 1984;25:133–7. 6363217 10.1136/gut.25.2.133 PMC1432247

[26] Gershwin ME Selmi C Worman HJ. Risk factors and comorbidities in primary biliary cirrhosis: a controlled interview-based study of 1032 patients. Hepatology (Baltimore, Md) 2005;42:1194–202. 10.1002/hep.20907 PMC3150736 16250040

[27] Howel D Fischbacher CM Bhopal RS. An exploratory population-based case-control study of primary biliary cirrhosis. Hepatology (Baltimore, MD) 2000;31:1055–60. 10.1053/he.2000.7050 10796879

[28] Parikh-Patel A Gold EB Worman H. Risk factors for primary biliary cirrhosis in a cohort of patients from the United States. Hepatology (Baltimore, MD) 2001;33:16–21. 10.1053/jhep.2001.21165 11124815

[29] Prince MI Ducker SJ James OF. Case-control studies of risk factors for primary biliary cirrhosis in two United Kingdom populations. Gut 2010;59:508–12. 20332522 10.1136/gut.2009.184218

